# Disrupted Sensory–Motor Network Integrity in Excessive Online Gaming: Insight From Multimodal Morphometric Mapping

**DOI:** 10.1002/brb3.71823

**Published:** 2026-10-08

**Authors:** Zulalanmu Abudouxukuer, Michael Afiadenyo, Zhenzhen Yang, Benjamin Becker, Stefania Ferraro, Elijah Agoalikum, Seth Kwadjo Angmorterh, Francis Tanam Djankpa, Latif Adams, Bharat B. Biswal, Benjamin Klugah‐Brown

**Affiliations:** ^1^ The Clinical Hospital of Chengdu Brain Science Institute, MOE Key Laboratory for Neuroinformation, Center for Information in Medicine, School of Life Science and Technology University of Electronic Science and Technology of China Chengdu China; ^2^ School of Psychological Sciences Monash University Clayton Victoria Australia; ^3^ State Key Laboratory of Brain and Cognitive Sciences The University of Hong Kong Hong Kong China; ^4^ Department of Psychology The University of Hong Kong Hong Kong China; ^5^ China‐Cuba Belt and Road Joint Laboratory on Neurotechnology and Brain‐Apparatus Communication School of Life Science and Technology University of Electronic Science and Technology of China Chengdu China; ^6^ Key Laboratory of Psychosomatic Medicine Chinese Academy of Medical Sciences Chengdu China; ^7^ Sichuan Provincial Center for Mental Health Sichuan Provincial People's Hospital, School of Medicine University of Electronic Science and Technology of China Chengdu China; ^8^ Department of Medical Imaging School of Allied Health Sciences University of Health and Allied Sciences Ho Ghana; ^9^ Department of Physiology School of Medical Sciences University of Cape Coast Cape Coast Ghana; ^10^ Department of Microbiology and Immunology, School of Medical Sciences, College of Health and Allied Sciences University of Cape Coast Cape Coast Ghana; ^11^ Department of Biomedical Engineering New Jersey Institute of Technology Newark New Jersey USA

**Keywords:** gray matter networks, internet gaming disorder, serotonin–dopamine coupling, source‐based morphometry

## Abstract

**Introduction:**

Excessive online gaming (EOG) is a transitional stage between recreational gaming and internet gaming disorder (IGD), characterized by impaired self‐control and escalating engagement. Early identification of neural and behavioral vulnerabilities is crucial for understanding IGD development.

**Methods:**

Fifty‐nine young adults (30 EOG, 29 controls) completed structural MRI and behavioral assessments, including the IGD Scale‐Short Form (IGDS9‐SF) and the Liebowitz Social Anxiety Scale (LSAS). Source‐based morphometry (SBM) identified independent gray matter networks, while behavioral profiling and receptor‐level mapping examined functional and neurochemical associations.

**Results:**

SBM revealed six gray matter components, with one network encompassing the right thalamus and precentral gyrus showing reduced loading in EOG, which negatively correlated with IGDS9‐SF scores. Behavioral annotation linked this network to sensory integration, attention, and motor‐executive control. Spatial overlap with serotonergic and dopaminergic receptor distributions indicated potential neurotransmitter involvement.

**Conclusion:**

Early alterations in sensory‐motor networks and associated neurotransmitter systems may contribute to impaired self‐regulation and heightened reward sensitivity, providing a multimodal framework for understanding the progression from excessive gaming to IGD.

## Graphical abstract

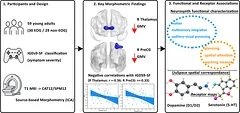

## 1 Introduction

With the global expansion of online gaming as a mainstream recreational activity, concerns have grown about its potential to shape maladaptive behaviors, particularly among young adolescents. Excessive and uncontrolled gaming has been associated with functional impairments in academic, social, and occupational domains (Chou et al. 2017; Kuss 2013), prompting formal recognition of internet gaming disorder (IGD) as a behavioral addiction. The Diagnostic and Statistical Manual of Mental Disorders, Fifth Edition (DSM‐5), listed IGD as a condition warranting further study (American Psychiatric Association 2013), while the International Classification of Diseases (ICD) includes “gaming disorder” under the category of addictive behaviors (World Health Organization 2018). The core symptoms of IGD include loss of behavioral control, prioritizing of gaming over other life goals, and continued engagement in gaming despite negative consequences (Bottel et al. 2023).

Moreover, a significant number of individuals exhibit persistent gaming‐related dysfunctions as a result of excessive use of online gaming platforms, leading to IGD (Tei and Fujino 2025). These excessive online gaming (EOG) individuals represent a high‐risk population between normative gaming and clinical addiction. Their behavioral characteristics, including impaired control, gaming prioritization, and continuation despite adverse consequences, indicate an emerging neurobiological vulnerability that may precede the onset of IGD, as assessed by the Internet Gaming Disorder Scale‐Short Form (IGDS9‐SF) (Pontes and Griffiths 2015). Several studies have employed similar criteria while individuals with a score ≥ 21 and exhibiting functional impairments as problematic online gamers (Monacis et al. 2016; Şalvarlı and Griffiths 2019). This group not only demonstrates excessive gaming behavior but also exhibits pronounced social dysfunction, making them a critical target for studying the transitional stage toward IGD and for informing the implementation of early intervention strategies.

Neuroimaging research has elucidated multiple neural correlates of IGD, revealing structural and functional alterations in networks mediating reward processing, cognitive control, and sensory‐motor integration(Kuss et al. 2018). Functional alterations, gray matter reductions, and altered white matter integrity have been observed in the prefrontal cortex, anterior cingulate, striatum, and thalamus (Han et al. 2012; Klugah‐Brown et al. 2021, 2022; Niu et al. 2022; Wang et al. 2015; Weng et al. 2013; Yuan et al. 2011; X. Zhou et al. 2020). These abnormalities converge on dysregulation within the cortico‐striato‐thalamo‐cortical (CSTC) circuits, which play a central role in habit formation and compulsive behaviors (A. Weinstein and Lejoyeux 2020). In addition, evidence from dopaminergic dysfunction models in substance and behavioral addictions suggests that alterations in mesothalamic dopamine pathways can increase sensitivity to salient cues and impair reinforcement control (Volkow et al. 2011; A. Weinstein and Lejoyeux 2020). However, research on high‐risk populations, such as individuals with EOG, remains limited, and findings on brain functional and structural alterations during the early, non‐pathological stages of excessive gaming have been inconsistent (Yu et al. 2022, 2021; F. Zhou et al. 2019), particularly with respect to subtle structural brain changes.

To address this gap, a cross‐dimensional morphometric approach, integrating source‐based morphometry (SBM) analysis, behavioral annotation, and spatial correlations with neurochemical systems could provide a multidimensional characterization of the neurobiological features underlying EOG. Traditional voxel‐based morphometry (VBM) approaches have provided valuable regional insights but are limited in capturing distributed, covarying patterns of gray matter variation that reflect system‐level integrity. SBM, a multivariate, independent component analysis (ICA)‐based technique, offers a data‐driven framework for identifying spatially covarying gray matter networks (Calhoun and Sui 2016; Xu et al. 2009). This method provides enhanced sensitivity to subtle, coordinated structural alterations compared with mass‐univariate VBM analyses and aligns with the growing emphasis on network‐level models of brain pathology (Sui et al. 2018). Recent advances have emphasized cross‐dimensional mapping between structural networks, behavioral phenotypes, and neurochemical systems. Spatial correlation of MRI‐derived morphometric changes with normative positron emission tomography (PET) and single‐photon emission computed tomography (SPECT) receptor atlases enables inferences about neurotransmitter‐related vulnerability (Dukart et al. 2021; Hansen et al. 2022). Moreover, recent studies on both substance use disorders (Yang et al. 2025) and behavioral addictions (Seo et al. 2020) have shown the critical interaction of certain neurotransmitters involved in these disorders. This approach potentially bridges macrostructural abnormalities with molecular‐level mechanisms, yielding a more mechanistic understanding of brain‐behavior associations.

In this study, we applied a multimodal framework integrating SBM, behavioral characterization, and receptor‐level mapping to investigate structural signatures in individuals with EOG compared with non‐EOG controls. Both the serotonergic and dopaminergic systems are deeply involved in reward processing, motivation, and executive control, which are core mechanisms underlying addictive behaviors. Dopamine within CSTC loops mediates reward salience and reinforcement learning (Haber and Knutson 2010), whereas serotonin modulates impulse control, behavioral inhibition, and affective regulation through its projections to the prefrontal cortex and striatum (Cools et al. 2008; Dalley and Roiser 2012). Dysregulation of these transmitter systems has been repeatedly implicated in both substance‐use and behavioral addictions, including IGD (Kühn and Gallinat 2015; A. Weinstein and Lejoyeux 2020).

Accordingly, we hypothesized that EOG participants would show reduced gray‐matter integrity within CSTC‐related regions engaged in reward evaluation and cognitive control, and that these alterations would spatially correspond to serotonergic and dopaminergic receptor distributions, indicating transmitter‐specific vulnerability. At the network level, we further expected that these regions would participate in a broader circuit encompassing the CSTC pathways and their interactions with the default mode network (DMN), reflecting the imbalance between internally directed reward salience and externally oriented executive regulation that has been frequently reported in gaming and other addictions (Dong et al. 2012; Menon 2011).

## 2 Methods

### 2.1 Participant Inclusion Criteria

Fifty‐nine right‐handed young adults (30 EOG, 29 non‐EOG; age range: 18–35 years) participated in the study. All participants had normal or corrected‐to‐normal vision, no history of neurological or psychiatric disorders, and no current or past substance dependence, verified via structured screening. Participants were recruited from local universities through online advertisements. Inclusion criteria required absence of contraindications for magnetic resonance imaging (MRI). All participants provided written informed consent in accordance with the Declaration of Helsinki. The study protocol was approved by the Institutional Review Board of the University of Electronic Science and Technology of China (UESTC) (Approval No. 106142022030722657).

### 2.2 Pre‐Scanning Assessment

The severity of gaming‐related behaviors was assessed using the IGDS9‐SF (Pontes and Griffiths 2015). The scale includes nine items reflecting DSM‐5 IGD criteria, each rated on a 5‐point Likert scale (1 = “never,” 5 = “very often”), yielding total scores ranging from 9 to 45. Participants scoring ≥ 21 and endorsing at least five DSM‐5 criteria were classified as EOG (Monacis et al. 2016). Participants scoring below these thresholds were classified as non‐EOG.

Social anxiety was assessed using the Liebowitz Social Anxiety Scale (LSAS) (Liebowitz 1987). Based on prior findings that IGD symptom severity is associated with social anxiety (Klugah‐Brown et al. 2022), LSAS was included in this current study to examine potential differences in social anxiety between EOG and non‐EOG individuals.

Internal consistency reliability was assessed using Cronbach's *α*. The IGDS9‐SF demonstrated excellent internal consistency (*α* = 0.944). The LSAS showed excellent reliability (Total *α* = 0.975; Fear *α* = 0.959; Avoidance *α* = 0.949).

### 2.3 MRI Data Acquisition

MRI data were acquired using a 3.0 T MRI scanner (Discovery MR750, GE, USA) at the Information Medicine Research Center of the University of Electronic Science and Technology. The participants lay supine on the MRI couch, head‐first. Participants were provided with earplugs for hearing protection due to the loud noise associated with MRI scanning. Finally, participants’ heads were immobilized with foam pads and straps to minimize motion artifacts.

Whole‐brain T1‐weighted MR images were acquired using a fast gradient echo (MPRAGE) sequence with the following parameters: repetition time (TR) = 5.98 ms, echo time (TE) = 1.968 ms, flip angle = 12°, 156 slices, field of view (FOV) = 256 × 256 mm, and scanning matrix = 256 × 256, and voxel size = 1 × 1 × 1 mm^3^.

### 2.4 T1 Preprocessing

T1 images of all participants were preprocessed in MATLAB (R2022a) using the CAT12 toolbox (https://neuro‐jena.github.io/cat/) in SPM12 software (https://www.fil.ion.ucl.ac.uk/spm/). Specific procedures included bias correction, segmentation into gray matter, white matter, and cerebrospinal fluid images, spatial normalization to standard space using the DARTEL algorithm, and volumetric modulation of the gray matter and white matter images by Jacobi row patterns. Finally, the modulated gray‐ and white‐matter images were smoothed with a full‐width‐half‐maximum (FWHM) 8 mm Gaussian kernel to reduce noise and meet statistical analysis assumptions. Quality assurance metrics were inspected for sample homogeneity (CAT12 “sample homogeneity check”).

### 2.5 SBM Analysis

SBM was conducted using the Group ICA of fMRI Toolbox (GIFT) (https://trendscenter.org/software/gift/). First, the minimum description length (MDL) criterion was employed to estimate the number of independent components (Li et al. 2007), and the Infomax algorithm was applied for ICA (Bell and Sejnowski 1995). This algorithm minimizes mutual information among the output networks to identify naturally grouped, maximally independent sources. To ensure the robustness of the components, ICASSO was used to repeat the ICA analysis 20 times, followed by clustering (Himberg et al. 2004) to ensure component stability (Iq > 0.90 threshold). Subject‐specific loading coefficients were entered into two‐sample *t*‐tests within the GIFT framework to compare EOG and non‐EOG groups. Note that while second‐level modules in standard ICA/SBM toolboxes do not inherently support covariate adjustment during group‐wise spatial component contrasts, potential confounding effects of age, sex, and total intracranial volume (TIV) were directly accounted for in all subsequent dimensional and partial correlation analyses against behavioral severity metrics. Components surviving false discovery rate (FDR) correction at q<0.025 were retained. In contrast, behavioral relevance was examined; partial correlation analyses between component loadings and IGDS9‐SF scores were conducted across all participants, with age, sex, and TIV included as covariates. For regions contributing most strongly to differential components, regional gray matter volume (GMV) was extracted for post hoc partial correlation analyses with behavioral scores (IGDS9‐SF and LSAS), controlling for age, sex, and TIV, providing convergent evidence for component‐level findings. This approach aimed to verify whether the key brain regions constituting this differential network were individually associated with the behavioral scale, thereby validating the reliability of the network discovery results and identifying the core contributing brain regions.

### 2.6 Behavioral and Receptor‐Level Analysis

To functionally characterize the identified components, we used the Brain Annotation Toolbox (BAT) (Liu et al. 2019), referencing activation meta‐analyses from NeuroSynth. Behavioral terms significantly associated with the spatial extent of each component were identified using permutation testing (10,000 iterations; *q* < 0.05, FDR‐corrected). To explore whether the altered brain structural organization mirrors the neurotransmitter chemoarchitecture in EOG participants, spatial correlation analysis was employed to assess the spatial associations between abnormal brain patterns identified by SBM analysis and fourteen different neurotransmitter receptor/transporter maps provided by the JuSpace toolbox (Dukart et al. 2021). Receptor maps included dopaminergic (D1, D2, FDOPA, and DAT) (Alakurtti et al. 2015; Dukart et al. 2018; García‐Gómez et al. 2013; Jaworska et al. 2020; Kaller et al. 2017) and serotonergic (5‐HT1A, 5‐HT1B, 5‐HT2A, and SERT) (Beliveau et al. 2017; Fazio et al. 2016; Savli et al. 2012) systems. Specifically, the PET/SPECT‐derived neurotransmitter receptor/transporter maps provided by the JuSpace toolbox were registered to the standard MNI152 space. Because the SBM‐derived spatial maps were already represented in this same stereotactic space, voxel‐wise spatial correspondence was directly established between the two modalities. Neurotransmitter receptor and transporter distribution densities were then compared against the spatial distribution of GMV alterations derived from the SBM analysis. Subsequently, Fisher's *Z*‐transformed Pearson correlation coefficients were calculated between the receptor/transporter densities and the thresholded SBM spatial maps, with spatial autocorrelation adjusted via spatial partial correlation models. To control for multiple comparisons across the fourteen evaluated neurotransmitter maps, an FDR correction was applied at a significance threshold of q<0.05. This integrative analysis enabled a rigorous evaluation of whether regional GMV variations in key brain structures spatially mirrored the chemoarchitectural distribution patterns of specific neurotransmitter systems.

## 3 Results

### 3.1 Demographic and Behavioral Characteristics

There were no statistically significant differences in age, sex, education level, or LSAS scores between the 30 EOG and 29 non‐EOG participants (all *p* > 0.05). Consistent with the inclusion criteria, EOG participants recorded higher IGDS9‐SF scores, resulting in a statistically significant difference between the two groups (*p* < 0.001) (Table 1). Because LSAS scores did not significantly differ between groups, LSAS was not included as a covariate in the subsequent SBM analyses.

**TABLE 1:** Demographic information of the subjects.

	EOG (*N* = 30)	non‐EOG (*N* = 29)	*p*
Age	20.67 ± 2.19	21.55 ± 2.47	0.15
Education level	1.50 ± 0.51	1.30 ± 0.60	0.18
Gender (M/F)	19/11	22/7	0.30
IGDS9‐sf score	32.40 ± 5.02	16.33 ± 3.62	< 0.001
LSAS score	75.67 ± 29.01	62.83 ± 30.96	0.11

*Note*: Differences between groups were compared using two‐sample *t*‐tests or *χ* ^2^ tests (using distribution for gender).

### 3.2 Results of SBM Analysis

Based on the MDL criterion, we identified six reliable gray matter structural networks. Group comparison analysis revealed that only Component 6 exhibited a statistically significant difference in loading coefficients between the EOG and non‐EOG groups (*t* = −2.378, *p* = 0.02), with the EOG group showing lower loading coefficients, suggesting reduced GMV across the corresponding network regions. The spatial distribution pattern of this component was corrected for FDR (*q* < 0.025) (Figure 1a). Further correlation analysis showed a statistically significant association between the loading coefficient of Component 6 and IGDS9‐SF scores (Figure 1b). To control for multiple comparisons, we applied the Benjamini–Hochberg FDR correction across the three correlation analyses. The uncorrected results are provided in the Supporting Information (Figures S1 and S2). Further focusing on the most strongly contributing regions within Component 6, two representative clusters were extracted based on peak voxels |*Z*| > 5 and cluster size > 500 voxels, corresponding to the right thalamus (*X* = 10.5, *Y* = –15, *Z* = 7.5: R Thal) and the right precentral gyrus (*X* = 28.5, *Y* = –6, *Z* = 51: R PreCG). Regional GMV extracted from these clusters showed statistically significant negative correlations with IGDS9‐SF scores (Figure 2). In contrast, exploratory post hoc partial correlation analyses controlling for age, sex, and TIV revealed no significant associations between LSAS scores and either the Component 6 loading coefficient or regional GMV in the R Thal and R PreCG (all p>0.05, uncorrected). Given the absence of any significant relationships at the uncorrected threshold, correction for multiple comparisons was not applied to these analyses.

**FIGURE 1:**
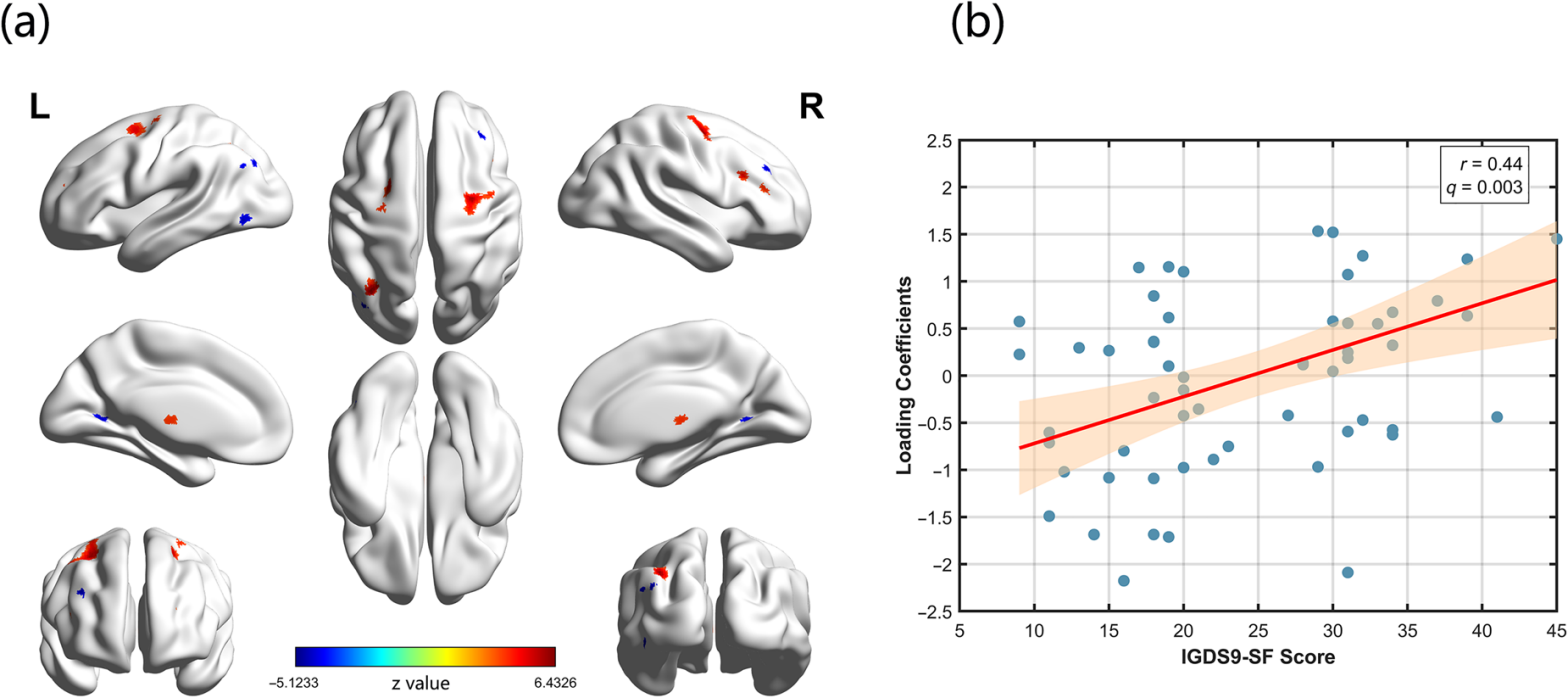
Spatial maps of components showing abnormal differences between the EOG and non‐EOG groups. (a) Spatial distribution of the loading coefficients of GMV Component 6. (b) The loading coefficients of Component 6 show a positive correlation with IGDS9‐SF scores.

**FIGURE 2:**
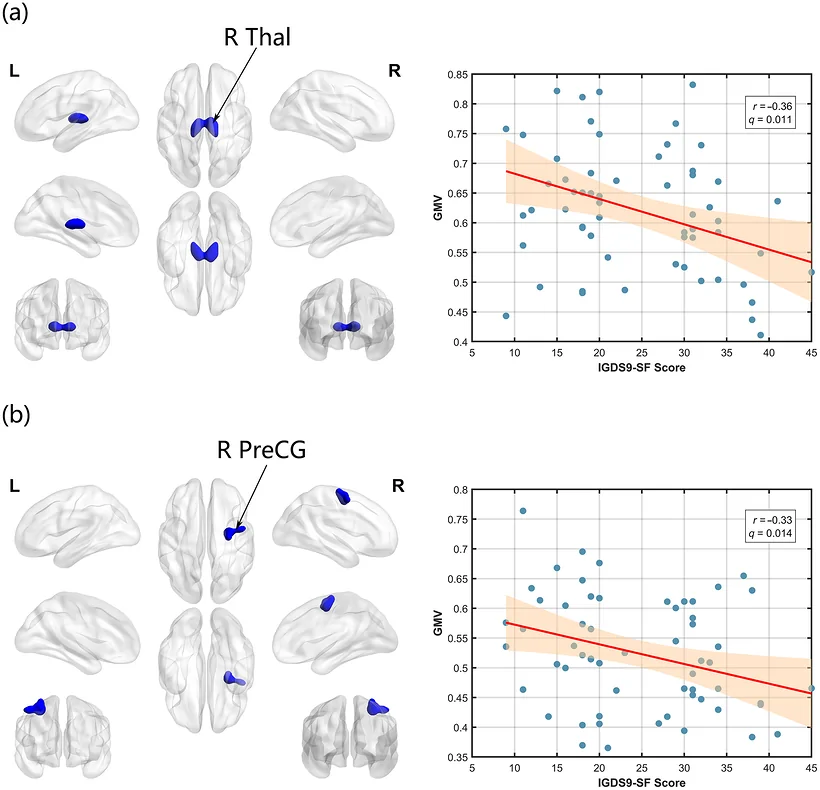
Results of the correlation analysis between the GMV of the two significant clusters of Component 6 and the IGDS9‐SF scores. (a) Negative correlation between the GMV of R Thal and IGDS9‐SF scores. (b) Negative correlation between the GMV of the right precentral gyrus (R PreCG) and IGDS9‐SF scores.

### 3.3 Behavioral and Receptor‐Level Analysis Results

The behavioral characterization analysis of the identified brain regions shows that the R Thal is significantly associated with motion, multisensory integration, auditory–visual processing, visual motion, perceptual processing, facial expressions, and dynamic visual stimuli (video), while the R PreCG is more closely related to planning, spatial attention, working memory, memory load, saccades, executive control, reasoning, monitoring, response selection, and visual information processing. Thus, structural changes in these two brain regions may affect game‐related sensory information processing, motor execution, and cognitive control processes, thereby influencing action coordination, attention allocation, and impulse control in gaming (Figure 3). The detailed functional annotation results are reported in Tables S1 and S2.

**FIGURE 3:**
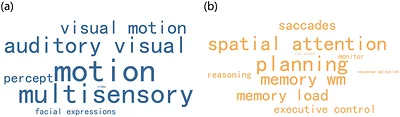
Functional characterization of the R Thal and R PreCG regions. (a) Word cloud of functional terms for the R Thal; (b) word cloud of functional terms for the R PreCG. Only terms surviving FDR correction (*q* < 0.05) are considered. For the R Thal, all FDR‐significant terms are displayed, whereas for the R PreCG, the top 10 terms ranked by activation percentile are shown for visualization clarity. The size of each term reflects its activation percentile, indicating the relative contribution of each function to the corresponding brain region.

The spatial correlation analysis revealed that GMV alterations in both the R Thal and R PreCG were significantly associated with the serotonin and dopamine systems (Figure 4). Specifically, GMV changes in the R Thal exhibited positive spatial correlations with the distribution densities of serotonin receptors, dopamine receptors, their transporters, and the dopamine precursor, suggesting that IGD patients showed more pronounced GMV reductions in thalamic regions characterized by lower receptor and transporter densities. In contrast, GMV changes in the R PreCG were negatively correlated with the densities of serotonin receptors and transporters, but positively correlated with the densities of dopamine receptors, transporters, and the dopamine precursor. The detailed results are reported in Tables S3 and S4.

**FIGURE 4:**
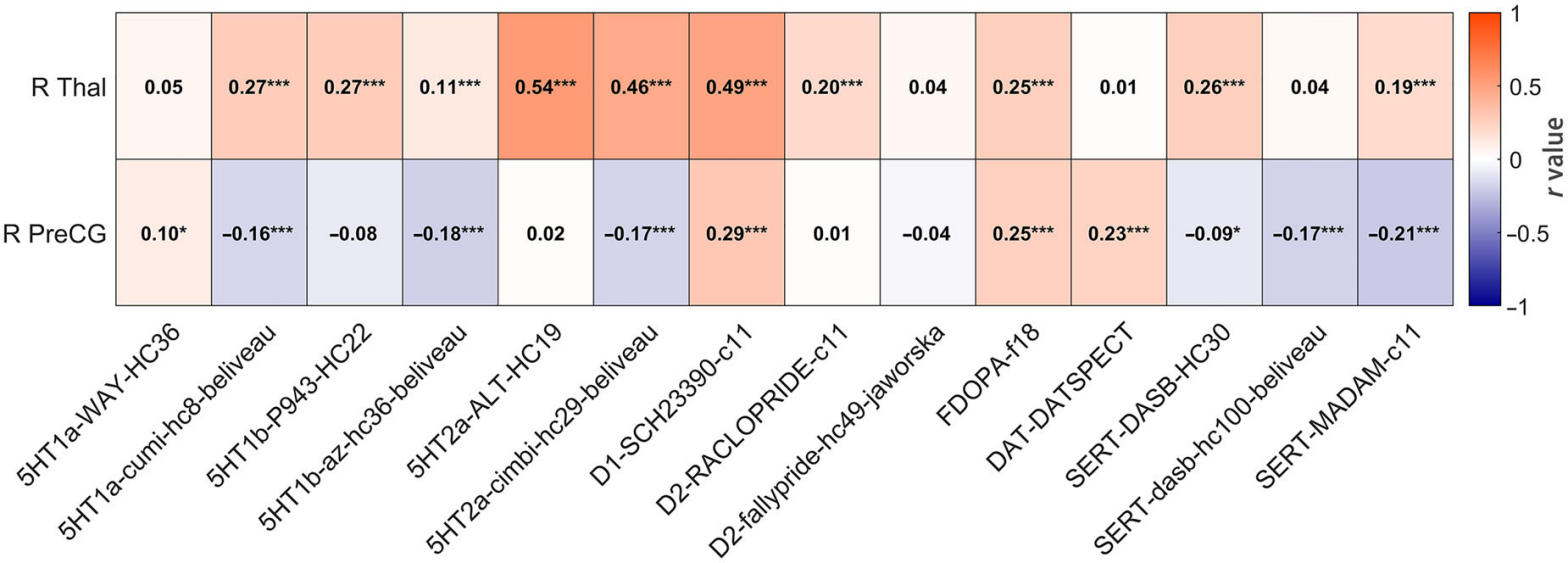
Spatial correlation between GMV variations in the R Thal and R PreCG and the densities of 14 neurotransmitter receptors/transporters. The neurotransmitter receptors/transporters are in order from left to right: serotonin receptors (5HT‐1A, 5HT‐1B, and 5HT‐2A), dopamine receptors (D1, D2), dopamine precursor (FDOPA), dopamine transporter (DAT), and serotonin transporter (SERT). These significant results all passed FDR correction, where *** indicates *q* < 0.001, ** indicates *q* < 0.01, and * indicates *q* < 0.05.

## 4 Discussion

### 4.1 Summary and Interpretation of Key Findings

This study employed both categorical and dimensional approaches combined with a sensitive morphometric strategy to examine brain structural alterations in individuals exhibiting EOG, a population at potentially high risk for developing IGD. The SBM analysis identified six structural components, of which one (C6) showed significantly lower loading coefficients in the EOG group than in non‐EOG participants. We acknowledge that the mean IGDS9‐SF score for the EOG group (32.40 ± 5.02) is relatively high, suggesting that some individuals may exhibit more severe or near‐clinical levels of gaming‐related symptoms. The inclusion of individuals with more severe symptoms may therefore amplify the observed neural and neurotransmitter differences, particularly in the spatial associations between GMV changes and neurotransmitter distribution. Lower component loadings, reflecting reduced gray matter integrity, were associated with higher IGDS9‐SF scores, indicating that participants with more severe gaming symptoms exhibited greater gray matter reduction within this network. This effect was primarily driven by the R Thal and R PreCG, both of which showed independent negative correlations between GMV and symptom severity. Functional annotation further revealed that these regions support sensory integration and executive‐motor control, and that their GMV alterations were spatially associated with specific serotonergic and dopaminergic receptor systems.

### 4.2 Thalamus and Sensory–Reward Integration and Precentral Gyrus in Executive–Motor Control

The thalamus serves as a key convergence hub, integrating sensory, cognitive, and reward‐related information to support adaptive behavior. Through reciprocal projections with the prefrontal cortex, striatum, and limbic structures, the thalamus regulates the relay of sensory input to cortical regions and modulates attentional allocation and motivational salience (Mitchell et al. 2014). This integrative role extends beyond passive transmission; it contributes actively to gating sensory signals and prioritizing reward‐predictive stimuli. Neurophysiological evidence shows that specific thalamic nuclei, including the mediodorsal and pulvinar nuclei, participate in regulating attention and cognitive control under motivational contexts and, as such, critically mediate context‐dependent inhibitory control (Haber and Calzavara 2009; Halassa and Kastner 2017; Zhuang et al. 2023).

In the context of behavioral addiction, repeated exposure to high‐reward visual stimuli and rapid reinforcement schedules may recalibrate thalamic sensory–reward pathways. Structural reductions in thalamic gray matter, as observed in the present study, likely reflect maladaptive plasticity arising from repeated engagement of sensory–reward circuits. Such reductions can disrupt thalamocortical gating, thereby enhancing the relative salience and attentional capture of gaming‐related cues over neutral stimuli (Kim et al. 2021; X. Zhou et al. 2025). Functional MRI studies consistently demonstrate heightened thalamic activation during exposure to game‐associated cues, coupled with stronger connectivity between the thalamus, ventral striatum, and orbitofrontal cortex (Dong, Zhou, and Zhao 2010; A. Weinstein and Lejoyeux 2020). This hyper‐responsiveness contributes to increased reward anticipation and craving, which are key behavioral markers of addiction.

Moreover, recent multimodal studies suggest that thalamic dysfunction in behavioral addictions mirrors findings in substance use disorders. For example, reduced thalamic volume and altered thalamostriatal connectivity have been linked to diminished top‐down control from the dorsolateral prefrontal cortex and enhanced sensitivity to conditioned rewards (Han et al. 2012; Niu et al. 2022; Solly et al. 2022). These convergent findings support the interpretation that GMV reductions in the thalamus may represent a structural correlate of impaired sensory‐reward integration, predisposing individuals to excessive motivational engagement with gaming stimuli.

The precentral gyrus, encompassing the primary motor cortex (M1) and parts of the premotor cortex, serves a dual role: executing voluntary movement and supporting higher‐order executive‐motor regulation. Beyond its classical motor functions, the precentral gyrus participates in motor imagery, inhibitory control, and cognitive flexibility (Jin et al. 2022; Tomasino and Gremese 2016). It forms part of the frontostriatal circuitry that integrates motor preparation with decision‐making and self‐regulation. The precentral and supplementary motor areas contribute to inhibitory control by suppressing prepotent motor impulses, particularly under conditions requiring delayed or context‐dependent responses (Duque et al. 2017; Simon et al. 2002). Gray matter reduction in this region, as observed in the EOG participants, may thus signify compromised inhibitory processing and action selection.

Similar alterations have been documented in individuals suffering from IGD and other impulse‐control disorders, where reduced precentral and dorsolateral prefrontal integrity correlates with diminished capacity to regulate motor impulses and attenuate cue‐triggered urges (Kühn and Gallinat 2015; Yuan et al. 2011). Resting‐state and task‐based fMRI studies further show that abnormal activity in motor and premotor regions during gaming tasks is associated with impaired motor planning, overlearned response patterns, and reduced executive control (Dong et al. 2019; A. M. Weinstein 2017). This neural profile aligns with behavioral findings showing excessive habitual responding and diminished cognitive flexibility in individuals with problematic gaming habits (Brand et al. 2019; Dong and Potenza 2014).

Importantly, both the thalamus and precentral gyrus are integral components of the CSTC loops, which underlie the dynamic interplay between motivation, cognition, and action (Alexander and Crutcher 1990; Haber and Calzavara 2009). Structural or functional perturbations within these loops can weaken the top‐down modulation of motor readiness by executive regions, thereby favoring compulsive, stimulus‐driven behaviors. In individuals involved with EOG, disrupted CSTC integration may manifest as increased motor preparedness to gaming cues, reduced cognitive inhibition, and a heightened drive for continuous engagement, a neural phenotype paralleling the transition from voluntary to compulsive gaming.

To more directly relate these structural findings to observable gaming behaviors, reduced thalamic integrity may contribute to heightened attentional capture by gaming‐related reward cues, resulting in greater difficulty disengaging from gameplay once initiated or increased susceptibility to re‐engagement following exposure to gaming‐associated visual or auditory stimuli. This interpretation is consistent with evidence indicating that the thalamus serves as a key node in CSTC circuits that integrate sensory information with motivational salience and contribute to cue‐induced craving and attentional bias across addictive disorders (Halassa and Kastner 2017; Volkow et al. 2016). In parallel, reduced gray matter integrity within the precentral gyrus may reflect compromised executive‐motor control, reducing the capacity to suppress prepotent motor responses and terminate gaming despite the intention to stop, a behavioral hallmark of impaired inhibitory control in IGD (Brand et al. 2019). Beyond its primary motor function, the precentral gyrus forms part of the broader fronto‐basal ganglia circuitry supporting motor preparation, response inhibition, and goal‐directed action selection, processes that are consistently disrupted in addictive behaviors (Duque et al. 2017). Future studies integrating structural neuroimaging with ecological momentary assessment (EMA) and validated behavioral paradigms, including cue‐reactivity and stop‐signal tasks, will be important for determining whether alterations within the thalamic‐precentral structural network predict real‐world attentional bias, inhibitory control deficits, and the progression of gaming‐related symptom severity.

Together, these findings highlight that thalamic sensory‐reward dysregulation and precentral executive‐motor impairment are interconnected facets of early addiction vulnerability. Reduced gray matter in these regions may signify a broader network‐level imbalance between reward sensitivity and inhibitory control, providing a structural substrate for the emergence of maladaptive gaming behavior.

### 4.3 Neurotransmitter Coupling Mechanisms

The observed structural alterations in the R Thal and R PreCG were both significantly coupled with the spatial organization of serotonergic and dopaminergic receptor systems, suggesting that these regions may constitute neurochemically sensitive hubs in the early stages of behavioral addiction. This coupling pattern provides insight into how large‐scale neurotransmitter gradients modulate the vulnerability of cortical and subcortical circuits to repetitive, reward‐linked behaviors such as online gaming.

#### 4.3.1 Thalamic Coupling and Neurochemical Vulnerability

The thalamus contains dense reciprocal projections with prefrontal, striatal, and limbic regions, serving as a key relay for sensory integration and reward learning (Mitchell et al. 2014; Wolff et al. 2021). Spatial mapping studies using PET and transcriptomic atlases demonstrate that thalamic subnuclei exhibit heterogeneous but generally lower serotonin (5‐HT) and dopamine (DA) receptor densities relative to cortical targets (Goulas et al. 2021; Hansen et al. 2022). In the current study, GMV reductions were most pronounced in thalamic territories characterized by sparse receptor and transporter distributions, consistent with the joint‐vulnerability hypothesis: brain regions with limited neuromodulatory capacity are less able to buffer repetitive reward stimulation and thus undergo earlier structural decline (Froudist‐Walsh et al. 2023; Zhao et al. 2023). Similar patterns have been observed in dopaminergic dysfunction models of substance and behavioral addictions, where reduced receptor availability in mesothalamic projections correlates with hypersensitivity to salient cues and impaired reinforcement control (Volkow et al. 2011; A. Weinstein and Lejoyeux 2020).

#### 4.3.2 Precentral Gyrus Coupling and Inhibitory Control Imbalance

The precentral gyrus integrates motor preparation with executive regulation via corticostriatal–thalamocortical loops (Alexander and Crutcher 1990). Its receptor architecture shows relatively high serotonergic innervation, particularly of 5‐HT1A and 5‐HT2A receptors, and moderate dopaminergic receptor expression (Beliveau et al. 2017; Kurose et al. 2021). The finding that GMV reduction was strongest in regions with high serotonin but low dopamine density implies an imbalance between inhibitory (serotonergic) and excitatory/motivational (dopaminergic) control. Serotonin modulates top‐down inhibition and behavioral restraint (Dalley and Roiser 2012), whereas dopamine facilitates action selection and reinforcement learning (Palmiter 2008). Disproportionate serotonergic influence, when not counterbalanced by dopamine, has been linked to excessive avoidance or repetitive engagement behaviors and reduced cognitive flexibility (Hansen et al. 2022; Palomero‐Gallagher and Zilles 2018). In the context of EOG, such neurochemical asymmetry may weaken executive monitoring while sustaining motor readiness toward game‐related stimuli, thereby promoting compulsive play.

#### 4.3.3 Systemic Interaction and Behavioral Expression

The behavioral characterization of the identified regions indicates distinct yet functionally complementary roles for the R Thal and R PreCG. The R Thal was significantly associated with perception, perceptual processing, face recognition, action, social interaction, and acoustic processing. The thalamus serves as a central relay integrating multisensory input and coordinating perceptual‐motor responses (Mitchell et al. 2014; Wolff et al. 2021). Structural disruption in this region may impair the efficiency of sensory information transfer to cortical areas, heightening attentional bias toward gaming‐related visual or auditory cues and strengthening perceptual engagement during play. Prior neuroimaging evidence shows that altered thalamic structure and connectivity are linked to enhanced cue reactivity and reward sensitivity in individuals with IGD (Han et al. 2012; Kim et al. 2021; W. R. Zhou et al. 2021).

In contrast, the R PreCG was associated with attention, working memory, action, cognitive control, and motor imagery, which are functions essential for the regulation of voluntary behavior and response inhibition. The precentral gyrus, encompassing primary and premotor cortices, contributes to motor readiness and executive‐motor integration (Jin et al. 2022; Tomasino and Gremese 2016). Reduced gray matter integrity in this region likely reflects diminished capacity for motor planning and cognitive control, impairing the ability to inhibit repetitive, cue‐driven behaviors that dominate gaming contexts. Functional MRI studies have demonstrated that abnormal activation within motor and premotor cortices corresponds with higher impulsivity and habitual response tendencies in individuals with problematic gaming or gambling behaviors (Kühn and Gallinat 2015; A. M. Weinstein 2017).

Thus, the co‐occurring structural changes in the thalamus and precentral gyrus may alter how game‐related sensory information is integrated, executed, and regulated at the behavioral level. Such alterations could bias attention allocation, action coordination, and impulse control, reinforcing the habitual, immersive engagement characteristic of EOG and representing an early behavioral signature of vulnerability toward IGD.

Importantly, this interpretation is further supported by the distinct double dissociation observed between gaming symptom severity and social anxiety. Whereas the Component 6 loading coefficients and regional GMV within the R Thal and R PreCG exhibited significant negative correlations with IGDS9‐SF scores, no such phenotypic relationships emerged with LSAS scores, even after robustly controlling for age, sex, and TIV. This divergent statistical pattern may imply that the observed macrostructural gray matter reductions within this thalamic‐precentral circuit are selectively coupled to the severity of gaming‐related behavioral metrics rather than a generalized manifestation of social anxiety. Although clinical literature frequently posits social anxiety as a core comorbid or predisposing vulnerability factor for problematic online gaming, this clear dissociation suggests that the identified network shifts are more specific to gaming‐centric neuroplastic changes or behavioral adaptations rather than a broader, shared affective or social vulnerability phenotype.

### 4.4 Limitations and Future Directions

Several limitations warrant consideration, each highlighting specific avenues for future research. First, the cross‐sectional design precludes causal inferences regarding whether observed structural abnormalities (e.g., GMV reductions in the right thalamus and precentral gyrus) are predisposing vulnerabilities or neuroplastic consequences of EOG. Future longitudinal or interventional studies tracking neural trajectories are needed to establish causal directionality. Second, reliance on a homogeneous university sample classified via self‐report limits external validity. While this minimized confounding variance related to education and age, it precludes definitive clinical IGD diagnosis and restricts generalizability to other developmental stages or more severe clinical populations. Future investigations should employ lifespan designs and multi‐site cohorts using standardized diagnostic interviews (e.g., Structured Clinical Interview for DSM‐5) to map these alterations across the full subclinical‐to‐clinical spectrum. Third, the modest sample size (*N* = 59) limits statistical power, potentially obscuring subtle network alterations beyond the significant Component 6. Subsequent research requires larger cohorts to validate the reproducibility of these network‐level effects. Fourth, although social anxiety (LSAS) did not confound our primary metrics, the absence of comprehensive assessments for depression, impulsivity, or general intelligence leaves potential residual cognitive or affective confounding. Future work must integrate exhaustive neuropsychological and psychiatric phenotyping to isolate gaming‐specific neural effects from comorbid traits. Nevertheless, despite these limitations, the multimodal convergence of SBM‐derived structural networks, behavioral metrics, and receptor‐density profiles underscores the neurobiological robustness and specificity of our core observations.

## 5 Conclusion

EOG individuals exhibited reduced integrity of a gray matter structural network encompassing the R Thal and R PreCG. Network integrity was inversely related to gaming symptom severity and spatially aligned with distributions of serotonergic and dopaminergic receptors. These results suggest that early alterations in sensory‐motor integration networks, coupled with neurotransmitter system imbalances, may represent a neural vulnerability marker preceding the onset of clinical IGD. The findings provide a preliminary multilevel model linking gray matter architecture and neurochemical modulation in the early stages of gaming‐related addictive behavior.

## Author Contributions

**Michael Afiadenyo**: validation. **Benjamin Becker**: writing – review and editing, validation. **Seth Kwadjo Angmorterh**: writing – review and editing, validation. **Zhenzhen Yang**: software, validation. **Francis Tanam Djankpa**: writing – review and editing, validation. **Zulalanmu Abudouxukuer**: formal analysis, visualization, writing – original draft, software, investigation, methodology. **Stefania Ferraro**: writing – review and editing, validation. **Latif Adams**: writing – review and editing, validation. **Bharat B. Biswal**: writing – review and editing, validation. **Benjamin Klugah‐Brown**: conceptualization, methodology, validation, funding acquisition, writing – review and editing, supervision, project administration, resources. **Elijah Agoalikum**: writing – review and editing, validation.

## Funding

This study was supported by the China MOST2030 Brain Project (Grant No. 2022ZD0208500), and we are grateful for their support.

## Ethics Statement

All participants provided written informed consent in accordance with the Declaration of Helsinki. The study protocol was approved by the Institutional Review Board of the University of Electronic Science and Technology of China (UESTC) (Approval No. 106142022030722657).

## Conflicts of Interest

The authors declare no conflicts of interest.

## Supporting information




**Supplementary Materials**: brb371823‐sup‐0001‐SuppMat.docx

**Supplementary Materials**: brb371823‐sup‐0002‐S1‐S4.docx

## Data Availability

The data that support the findings of this study are available from the corresponding author upon reasonable request.
