# Supplementary material for: Disrupted Sensory–Motor Network Integrity in Excessive Online Gaming: Insight From Multimodal Morphometric Mapping
Source: Brain Behav. 2026 Oct 8;16(10):e71823. doi: 10.1002/brb3.71823 (PMC13650394; doi:10.1002/brb3.71823)
Supplement: Supplementary file 1 [file BRB3-16-e71823-s001.docx]

**Supplementary Resuls**

Figure S 1. Spatial maps of components showing abnormal differences between the EOG and non-EOG groups (uncorrected results, before Benjamini–Hochberg FDR correction).

(a) Spatial distribution of the loading coefficients of GMV component 6.

(b) The loading coefficients of component 6 show a positive correlation with IGDS9-SF scores.

Figure S 2. Results of the correlation analysis between the GMV of the two significant clusters of component 6 and IGDS9-SF scores (uncorrected results, before Benjamini–Hochberg FDR correction). (a) Negative correlation between the GMV of the right thalamus (R Thal) and IGDS9-SF scores. (b) Negative correlation between the GMV of the right precentral gyrus (R PreCG) and IGDS9-SF scores.

Figure S 3. Violin plots showing the distribution of LSAS (Liebowitz Social Anxiety Scale) scores in the EOG and non-EOG groups. Shapiro–Wilk tests confirmed normality in both groups (EOG: W = 0.968, p = 0.495; non_EOG: W = 0.950, p = 0.183).
