# Supplementary material for: Disrupted Sensory–Motor Network Integrity in Excessive Online Gaming: Insight From Multimodal Morphometric Mapping
Source: Brain Behav. 2026 Oct 8;16(10):e71823. doi: 10.1002/brb3.71823 (PMC13650394; doi:10.1002/brb3.71823)
Supplement: Supplementary file 2 [file BRB3-16-e71823-s002.docx]

Table S 1: Functional annotation results of R Thal from BAT analysis.

| Term Name | Activation Percentilie | Permute p | q |
| --- | --- | --- | --- |
| motion | 0.120148277 | <0.0001 | <0.0001 |
| multisensory | 0.085150458 | <0.0001 | <0.0001 |
| auditory visual | 0.077278674 | <0.0001 | <0.0001 |
| visual motion | 0.053881378 | <0.0001 | <0.0001 |
| percept | 0.051297427 | <0.0001 | <0.0001 |
| facial expressions | 0.045540776 | <0.0001 | <0.0001 |
| video | 0.043643698 | <0.0001 | <0.0001 |
| perception | 0.16377017 | 0.0300 | 0.23250 |
| perceptual | 0.115721762 | 0.0200 | 0.18870 |
| face | 0.099520279 | 0.0300 | 0.23250 |

Table S 2: Functional annotation results of R PreCG from BAT analysis.

| Term Name | Activation Percentilie | Permute p | q |
| --- | --- | --- | --- |
| planning | 0.046478683 | <0.0001 | <0.0001 |
| spatial attention | 0.043656997 | <0.0001 | <0.0001 |
| memory wm | 0.043417675 | <0.0001 | <0.0001 |
| memory load | 0.03851157 | <0.0001 | <0.0001 |
| saccades | 0.035635844 | <0.0001 | <0.0001 |
| executive control | 0.0329454 | <0.0001 | <0.0001 |
| reasoning | 0.032127072 | <0.0001 | <0.0001 |
| monitor | 0.027402389 | <0.0001 | <0.0001 |
| response selection | 0.026055237 | <0.0001 | <0.0001 |
| visual information | 0.025503252 | <0.0001 | <0.0001 |

| Neurotransmitter | r | p | FDR |
| --- | --- | --- | --- |
| 5HT1a_WAY_HC36 | 0.054299741 | 0.0443 | FALSE |
| 5HT1a_cumi_hc8_beliveau | 0.27250254 | <0.0001 | TRUE |
| 5HT1b_P943_HC22 | 0.267499526 | <0.0001 | TRUE |
| 5HT1b_az_hc36_beliveau | 0.114067968 | <0.0001 | TRUE |
| 5HT2a_ALT_HC19 | 0.538456128 | <0.0001 | TRUE |
| 5HT2a_cimbi_hc29_beliveau | 0.459802972 | <0.0001 | TRUE |
| D1_SCH23390_c11 | 0.486804238 | <0.0001 | TRUE |
| D2_RACLOPRIDE_c11 | 0.201027433 | <0.0001 | TRUE |
| D2_fallypride_hc49_jaworska | 0.043413788 | 0.1078 | FALSE |
| FDOPA_f18 | 0.252333043 | <0.0001 | TRUE |
| DAT_DATSPECT | 0.011943672 | 0.6581 | FALSE |
| SERT_DASB_HC30 | 0.255829815 | <0.0001 | TRUE |
| SERT_dasb_hc100_beliveau | 0.03564207 | 0.1867 | FALSE |
| SERT_MADAM_c11 | 0.190591209 | <0.0001 | TRUE |

Table S 3: Spatial correlation analysis between the SBM-derived R Thal cluster and neurotransmitter receptor density maps.

Table S 4: Spatial correlation analysis between the SBM-derived R PreCG cluster and neurotransmitter receptor density maps.

| Neurotransmitter | r | p | FDR |
| --- | --- | --- | --- |
| 5HT1a_WAY_HC36 | 0.099 | 0.0170 | TRUE |
| 5HT1a_cumi_hc8_beliveau | -0.164446963 | <0.0001 | TRUE |
| 5HT1b_P943_HC22 | -0.082 | 0.0480 | FALSE |
| 5HT1b_az_hc36_beliveau | -0.183148649 | <0.0001 | TRUE |
| 5HT2a_ALT_HC19 | 0.022 | 0.5893 | FALSE |
| 5HT2a_cimbi_hc29_beliveau | -0.166 | <0.0001 | TRUE |
| D1_SCH23390_c11 | 0.294 | <0.0001 | TRUE |
| D2_RACLOPRIDE_c11 | 0.008 | 0.8403 | FALSE |
| D2_fallypride_hc49_jaworska | -0.038 | 0.3559 | FALSE |
| FDOPA_f18 | 0.249 | <0.0001 | TRUE |
| DAT_DATSPECT | 0.228 | <0.0001 | TRUE |
| SERT_DASB_HC30 | -0.086 | 0.0379 | FALSE |
| SERT_dasb_hc100_beliveau | -0.171900191 | <0.0001 | TRUE |
| SERT_MADAM_c11 | -0.209 | <0.0001 | TRUE |
